# Editorial: Understanding the role of head and body movement when navigating a complex auditory scene

**DOI:** 10.3389/fnins.2023.1340393

**Published:** 2023-12-20

**Authors:** Erol J. Ozmeral, Nathan C. Higgins

**Affiliations:** Department of Communication Sciences and Disorders, University of South Florida, Tampa, FL, United States

**Keywords:** spatial hearing, hearing impairment, head movement, auditory scene analysis, eye gaze, audiovisual (AV) feedback

In complex listening situations, speech understanding can be highly challenging due to multiple sound sources from multiple locations, background noise, and reverberation. Changes in head and body position while further complicating the acoustic environment, can also provide additional cues to understanding or directing a conversation. For example, recognizable head and eye gestures can convey listener understanding prior to a talker completing a sentence or for a talker to emphasize a point. Such body and head movements also play a major role in the peripheral acoustic cues available to the listener, such as the binaural and spectral changes critical to sound source localization and segregation. Despite our comprehensive understanding of many of these peripheral cues in isolation, traditional laboratory settings often seek to control for or eliminate the influences of head, body, and eye movements during experiments on auditory perception and speech communication.

The scope of this Research Topic was intended to broadly capture the impact that head, body, and eye movements have on everyday communication, sound perception, and navigation. This collection of research was seen as fundamental to understanding speech communication and auditory perception as it exists outside the laboratory, and accumulating knowledge will advance innovation and development in assistive hearing devices, virtual reality, and other clinical and consumer technology sectors that integrate across multiple sensory domains to provide ecologically centered user experiences. The present Research Topic with four articles provides an introduction to the many interactions that exist between sensory modalities: one study focuses on the visual coherence that improves speech listening (Yuan et al.); one study focuses on how audition can influence fine-motor skills (Guo et al.); one study explores the role of micro head movements in solving front-back confusions (McLachlan et al.); and finally, the Research Topic includes a review on the state-of-the-field as it relates to hearing and head movements (Higgins et al.).

The review article *Head movement and its relation to hearing* by Higgins et al. sets the stage for much of the Research Topic by providing a broad overview of the state-of-the-field. The authors begin by describing the physical parameters involved with natural movements, and then we get an introduction to the latest developments in communication and hearing technology that are affected by head and body movement.

Guo et al. in a paper titled *Effects of auditory feedback on fine motor output and the corticomuscular coherence during a unilateral finger pinch task*, provides a valuable perspective by showing how the relationship between body motion and hearing is a two-way street. In their study, the authors use behavioral and physiological methods to test whether auditory feedback can modulate fine motor skills. Their study confirms that not only do participants perform better on the task with auditory feedback, but there is also an observed modulatory effect in cortex that is associated with auditory feedback, perhaps opening the door to more effective rehabilitation and training strategies for persons with movement disorders.

In Yuan et al.'s article *The impact of temporally coherent visual cues on speech perception in complex auditory environments*, a simple but effective visual cue is used to mimic the potential lip movements that aide our perception of speech in complex acoustic scenes. For intermediate signal-to-noise ratios, the authors report an observed benefit of coherent visual stimuli when listeners are asked to transcribe masked speech.

Finally, in *Dynamic spectral cues do not affect human sound localization during small head movements* by McLachlan et al., the authors report on a localization study in which small head rotations in the yaw rotation axis provide substantial benefit in solving front-back confusions, but otherwise see no benefit from movement in other axes (e.g., pitch) regardless of available spectral cues.

Together, the present Research Topic illustrates how our understanding of the auditory system can be swayed by interactions with other sensory modalities (e.g., vision, motor, etc.). As we navigate in complex acoustic scenes, our ability to segregate auditory objects relies not only on the saliency of source features but also on our relative position in the world to that source. Do we have visual feedback to better comprehend speech? Do we have auditory feedback to better perform motor tasks? These are a sample of the questions that seek to explore the multisensory integration provides the foundation to interpreting the world around us, and are fundamental to future therapeutic and technological innovations.

## Author contributions

EO: Writing—original draft, Writing—review & editing. NH: Writing—review & editing.

